# Impact of diffusing lung capacity before and after neoadjuvant concurrent chemoradiation on postoperative pulmonary complications among patients with stage IIIA/N2 non-small-cell lung cancer

**DOI:** 10.1186/s12931-019-1254-0

**Published:** 2020-01-10

**Authors:** Sumin Shin, Yong Soo Choi, Jae Jun Jung, Yunjoo Im, Sun Hye Shin, Danbee Kang, Jong Ho Cho, Hong Kwan Kim, Jhingook Kim, Jae Ill Zo, Young Mog Shim, Keunchil Park, Myung-Ju Ahn, Yong Chan Ahn, Genehee Lee, Juhee Cho, Ho Yun Lee, Hye Yun Park

**Affiliations:** 1 Department of Thoracic and Cardiovascular Surgery, Samsung Medical Center, Sungkyunkwan University School of Medicine, Seoul, Republic of Korea; 2 Division of Pulmonary and Critical Care Medicine, Department of Medicine, Samsung Medical Center, Sungkyunkwan University School of Medicine, Seoul, Republic of Korea; 3 Samsung Advanced Institute for Health Sciences & Technology (SAIHST), Sungkyunkwan University, Seoul, Republic of Korea; 4 Division of Hematology-Oncology, Department of Medicine, Samsung Medical Center, Sungkyunkwan University School of Medicine, Seoul, Republic of Korea; 5 Department of Radiation Oncology, Samsung Medical Center, Sungkyunkwan University School of Medicine, Seoul, Republic of Korea; 6 Patient-Centered Outcomes Research Institute, Samsung Medical Center, Seoul, Republic of Korea; 7 Department of Radiology and Center for Imaging Science, Samsung Medical Center, Sungkyunkwan University School of Medicine, Seoul, Republic of Korea

**Keywords:** Non-small cell lung Cancer, Outcomes, Dlco, Postoperative complication

## Abstract

**Background and objective:**

This study aims to evaluate the impact of diffusing capacity of the lung for carbon monoxide (DLco) before and after neoadjuvant concurrent chemoradiotherapy (CCRT) on postoperative pulmonary complication (PPC) among stage IIIA/N2 non-small-cell lung cancer (NSCLC) patients.

**Methods:**

We retrospectively studied 324 patients with stage IIIA/N2 NSCLC between 2009 and 2016. Patients were classified into 4 groups according to DLco before and after neoadjuvant CCRT; normal-to-normal (NN), normal-to-low (NL), low-to-low (LL), and low-to-very low (LVL). Low DLco and very low DLco were defined as DLco < 80% predicted and DLco < 60% predicted, respectively.

**Results:**

On average, DLco was decreased by 12.3% (±10.5) after CCRT. In multivariable-adjusted analyses, the incidence rate ratio (IRR) for any PPC comparing patients with low DLco to those with normal DLco before CCRT was 2.14 (95% confidence interval (CI) = 1.36–3.36). Moreover, the IRR for any PPC was 3.78 (95% CI = 1.68–8.49) in LVL group compared to NN group. The significant change of DLco after neoadjuvant CCRT had an additional impact on PPC, particularly after bilobectomy or pneumonectomy with low baseline DLco.

**Conclusions:**

The DLco before CCRT was significantly associated with risk of PPC, and repeated test of DLco after CCRT would be helpful for risk assessment, particularly in patients with low DLco before neoadjuvant CCRT.

## Summary at a glance

Patients with low DLco before CCRT were more likely to experience postoperative pulmonary complications (PPC) compared to patients with normal DLco. Reduction of DLco after CCRT also increased risk of having PPC among patients with low DLco before CCRT.

## Introduction

Treatment outcomes are unfavorable in patients with stage IIIA/N2 non-small-cell lung cancer (NSCLC) [1, 2], and the optimal therapeutic approaches for N2 disease remain controversial. Neoadjuvant concurrent chemoradiotherapy (CCRT) followed by surgical resection has been adopted to enhance local control and improve survival [3–6]. However, several studies have determined that aggressive surgical resection after neoadjuvant CCRT is associated with an increased risk of immediate postoperative complications, predominantly pulmonary morbidity and mortality [7]; thus, a select group of patients to reduce postoperative pulmonary complications (PPC) and mortality is necessary to achieve optimal outcomes after neoadjuvant CCRT followed by surgical resection.

Patients with impaired pulmonary function, assessed by forced expiratory volume in 1 s (FEV_1_) and the diffusing capacity of the lung for carbon monoxide (DLco), have an increased risk of pulmonary complications and poorer survival outcomes [8, 9]. In IIIA/N2 disease planning for neoadjuvant CCRT, as DLco is generally reduced after CCRT, the DLco loss after CCRT has been highlighted to predict PPC.

Nevertheless, there were a few studies evaluating DLco after neoadjuvant CCRT as a predictor of PPC [10–13], which were conducted in a small number of patients with inconsistent results for impact of DLco after CCRT on PPC. Thus, we aimed to confirm the DLco change after CCRT and to evaluate the impact of DLco before and after CCRT on PPC among the large number of patients with stage IIIA/N2 NSCLC. Additionally, we performed subgroup analysis based on the surgical extent.

## Patients and methods

### Study population

This is a retrospective cohort study. The data was obtained from the lung cancer registry at Samsung Medical Center from January 2009 to December 2016 and there were 333 patients completed induction CCRT followed by surgery with curative intent for histologically confirmed stage IIIA/N2 NSCLC and underwent spirometry and DLco before and after neoadjuvant CCRT. We excluded patients with interstitial lung disease (*n* = 3), limited resection (*n* = 3), and salvage operation (*n* = 6), resulting in 321 patients. This study was approved by the Institutional Review Board of Samsung Medical Center, which exempted the requirement for informed consent as we only used de-identified data retrieved from electronic medical records (IRB no. 2018–08–007-001).

### Measurements

#### Preoperative evaluation

All patients had histologically proven NSCLC with ipsilateral mediastinal nodal metastases confirmed by histological and/or cytological examination (mediastinoscopy, endobronchial ultrasound-guided transbronchial needle aspiration [EBUS-TBNA], Chamberlain incision or thoracoscopy) or by 18F-flurodeoxyglucose positron emission tomography (PET)/computed tomography (CT) scan. Patients were staged according to the seventh edition of the TNM classification [1].

Spirometry and DLco were performed using the Vmax 22 system (SensorMedics, Yorba Linda, CA, USA) according to criteria established by the American Thoracic Society/European Respiratory Society [14, 15]. Absolute values of DLco (mL/mmHg/min) were obtained, and the percentage of predicted values (% pred) were calculated using formula based on a representative Korean sample, which adjusted standard hemoglobin level [16].

**Fig. 1:**
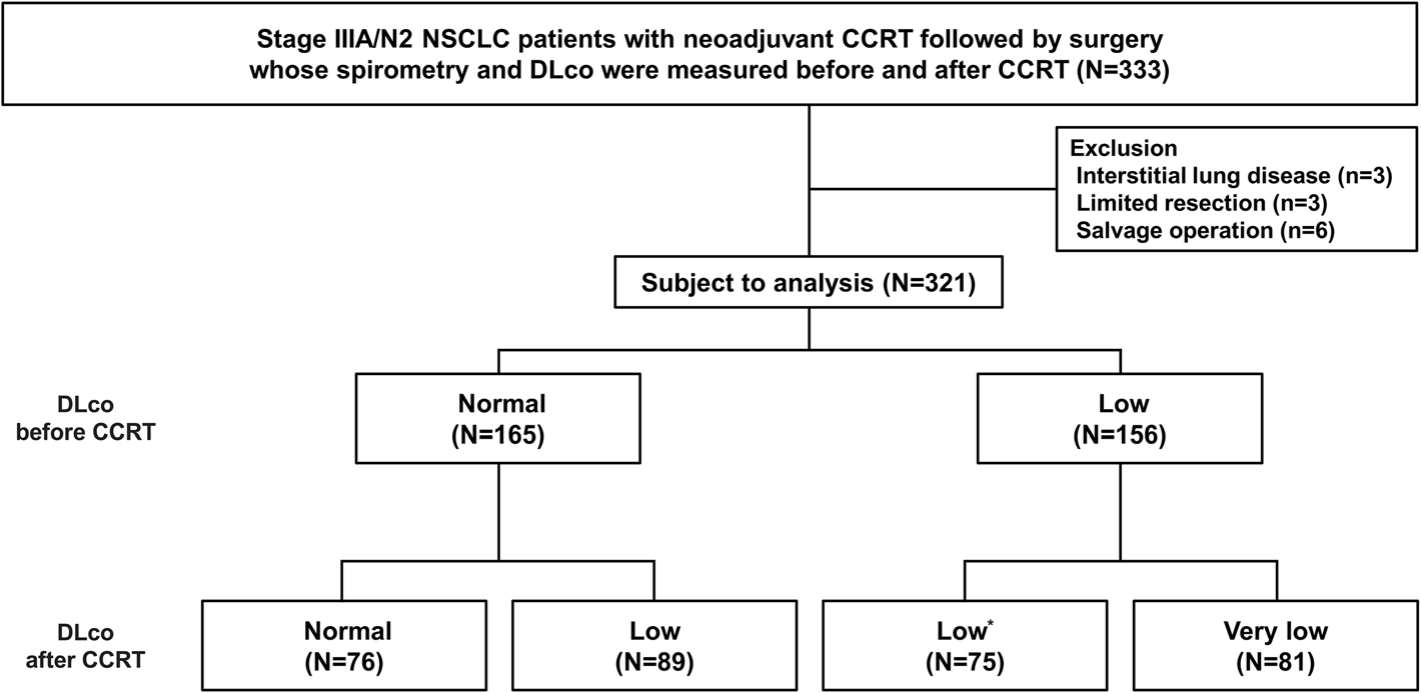
Study population. CCRT = concurrent chemoradiotherapy, NSCLC = non-small cell lung cancer, DLco = diffusing capacity of the lung for carbon monoxide. * This group include 8 patients whose DLco after CCRT was greater than 80%

Normal DLco was defined as DLco ≥80%, whereas low DLco was defined as DLco < 80% pred, [17]. Given that guideline suggest minimal requirement of postoperative DLco greater than 40% pred, very-low DLco was defined as DLco < 60% pred, which is moderate-to-severe DLco, % pred [18]. To evaluate the impact of change of DLco before and after CCRT on PPC, patients were classified into 4 groups based on DLco level before and after CCRT as follows; normal-to-normal (NN), normal-to-low (NL), low-to-low (LL), and low-to-very low (LVL). (Fig. 1).

The cardiopulmonary exercise test (CPET) was conducted in patients with the predicted postoperative FEV_1_ or DLco < 40% or to access performance status at the discretion of the treating surgeons. Of total 39 patients (12.1%) underwent CPET and all of them had greater than 15 ml/kg/min of maximal oxygen uptake (VO_2_max) except one 45-year young patient with 12.3 ml/kg/min.

#### Treatment scheme

Thoracic radiation therapy (TRT) was delivered to patients with a total dose of 44–45 Gy, 1.8 Gy/fraction over 5 weeks. The TRT target volume included the known gross and clinical disease plus adequate peripheral margins. The chemotherapy regimens consisted of weekly intravenous paclitaxel (50 mg/m^2^) or docetaxel (20 mg/m^2^) plus cisplatin (25 mg/m^2^) or carboplatin (AUC, 1.5) for 5 weeks. The first chemotherapy dose was delivered on the first day of TRT. Within 3 or 4 weeks following completion of neoadjuvant treatment, restaging procedures were performed with chest CT and/or PET/CT. Surgical resection was performed within4 to 6 weeks following the completion of neoadjuvant therapy unless the restaging workup showed evidence of progressive disease [6].

#### Postoperative pulmonary complications

PPC occurred during hospitalization or readmission during the first 60 days postoperatively were reviewed based on the medical records. PPC included pneumonia, acute respiratory distress syndrome (ARDS), respiratory failure, significant atelectasis requiring bronchoscopy or reintubation, bronchopleural fistula, empyema, and prolonged air leakage for more than 5 days. The PPC were classified according to the Clavien–Dindo classification [19], and complications exceeding grade II were analyzed. The definition of PPC is shown in Additional file 1: Table S1. The 30-day and 90-day postoperative mortality were also evaluated.

### Statistical analyses

Descriptive statistics were used to summarize the characteristics of patients by the incidence of PPC. Categorical variables were compared using the chi-square or Fisher’s exact tests, and continuous variables were compared using Student’s *t*-test. Comparison of pulmonary function tests before and after neoadjuvant CCRT were conducted using paired t-tests. Poisson regression with robust error variance was used to assess the association between DLco before and after neoadjuvant CCRT and PPC. For the main analyses, we calculated the multivariable-adjusted relative risk (aRR) and 95% confidence intervals (CI) of PPC. In addition, we modeled percent change as continuous variables using restricted cubic splines with knots at the 5th, 35th, 65th and 95th percentiles of the sample distribution to provide a flexible estimate of the dose-response relationship between percent change of DLco and PPC.

We used two models with increasing degrees of adjustment to account for potential confounding factors and to evaluate the role of potential biological mediators. Model 1 was adjusted for age at diagnosis, sex, and type of surgery, and model 2 was further adjusted for post-CCRT airflow limitation (FEV_1_ / FVC < 70%) and post-CCRT hemoglobin. We also performed sensitivity analyses for patients who underwent lobectomy to confirm the effects of DLco on PPC. All statistical analyses were two-sided with a significance level of 0.05. Analyses were performed using Stata software (ver.13.0; Stata Corp., College Station, TX, USA).

## Results

### Characteristics of study population

The mean age of the patients was 61.1 (8.0) and 80.7% of the study populations were male. Of total, 24.6% (*n* = 79) patients developed at least one PPC. Patient characteristics according to PPC were listed in Table 1. Patients with PPC were significantly older (63.3 year vs. 60.4 years) and more likely to be males (89.9% vs. 77.7%) and to have a smoking history (89.9% vs. 77.3%) compared to patients without PPC. Patients with PPC received more extensive resection, such as bilobectomy or pneumonectomy (30.4% vs. 16.9%), compared to those without PPC. Patients with PPC showed greater impairment in pulmonary function before CCRT, and their mean values of FEV_1_ (% pred) (*P* = 0.002) and DLco (% pred) (*P* < 0.001) were significantly lower than those without PPC.

**Table 1:** Characteristics of Study Populations by PPC Development (*N* = 321)

	Overall (*N* = 321)	Development of PPC		
		No (*N* = 242)	Yes (*N* = 79)	*P* Value
Age (years)	61.1 (8.0)	60.4 (8.0)	63.3 (8.0)	.006
Sex				.017
Male	289 (80.7)	188 (77.7)	71 (89.9)	
Female	62 (19.3)	54 (22.3)	8 (10.1)	
BMI (kg/m^2^)	23.7 (3.1)	23.8 (3.1)	23.6 (3.1)	.670
Smoking status				.014
Never	63 (19.6)	55 (22.7)	8 (10.1)	
Past or current	259 (80.4)	187 (77.3)	71 (89.9)	
Histology				.120
Adenocarcinoma	168 (52.3)	134 (55.4)	34 (43.0)	
Squamous cell	128 (39.8)	90 (37.2)	38 (48.1)	
Large cell	9 (2.8)	8 (3.3)	1 (1.3)	
Others	16 (5.0)	10 (4.1)	6 (7.6)	
Type of surgery				.010
Lobectomy	256 (79.8)	201 (83.1)	55 (69.6)	
Bilobectomy/Pneumonectomy	65 (20.2)	41 (16.9)	24 (30.4)	
PFT before CCRT				
FVC, % of the predicted value	91.7 (15.1)	92.7 (14.8)	88.6 (15.8)	.036
FEV_1_, % of the predicted value	82.8 (16.6)	84.4 (16.5)	77.8 (15.9)	.002
FEV_1_/FVC, %	70.2 (9.6)	70.9 (9.4)	68.1 (10.1)	.003
DLco, % of the predicted value	82.0 (17.5)	84.7 (17.7)	73.7 (14.1)	<.001
PFT after CCRT				
FVC, % of the predicted value	90.3 (13.7)	91.8 (13)	85.6 (14.8)	<.001
FEV_1_, % of the predicted value	84.4 (14.6)	85.9 (14.1)	79.8 (15.2)	.001
FEV_1_/FVC, %	70.8 (9.0)	71.2 (8.7)	69.5 (9.8)	.143
DLco, % of the predicted value	69.7 (15.1)	72 (14.9)	62.8 (13.6)	<.001
Hemoglobin				
Before CCRT	13.6 (1.4)	13.6 (1.4)	13.5 (1.6)	0.793
After CCRT	11.9 (1.5)	11.9 (1.5)	11.9 (1.6)	0.725

Values in table are mean (SD), number (%), or median (interquartile range)

*BMI* body mass index; *CCRT* concurrent chemoradiotherapy; *DLco* diffusing capacity of the lung for carbon monoxide; *FEV*_*1*_ forced expiratory volume in one second; *FVC* forced vital capacity; *PFT* pulmonary function test; *PPC* postoperative pulmonary complications

### Changes in pulmonary function after Neoadjuvant CCRT

**Fig. 2:**
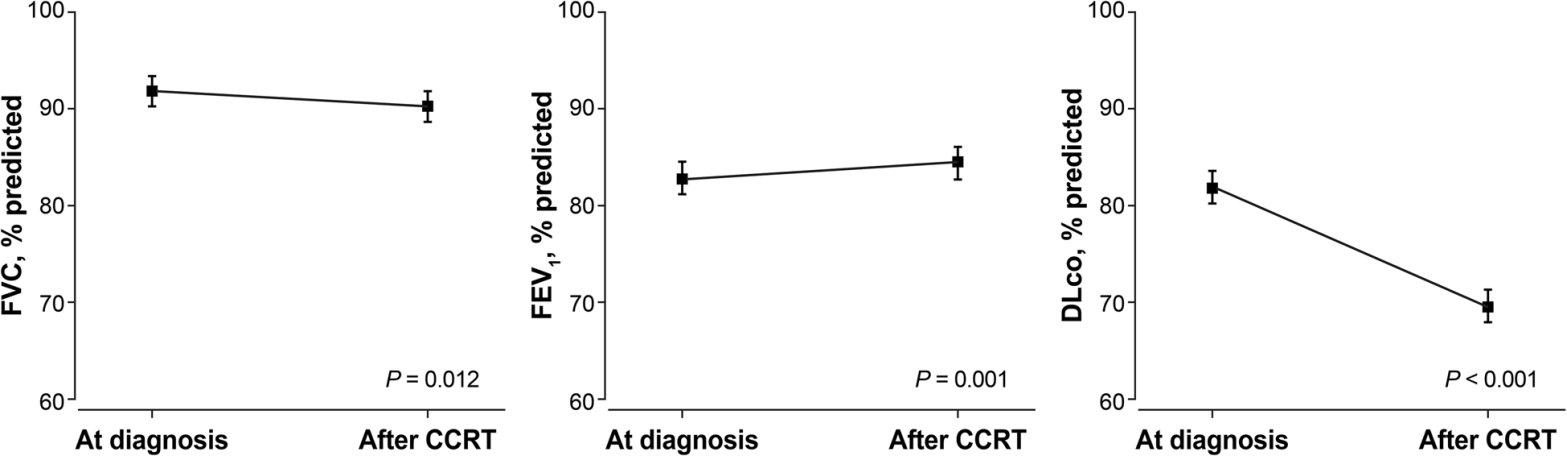
Change of pulmonary function after neoadjuvant CCRT. Neoadjuvant concurrent chemoradiotherapy (CCRT) was associated with a significant worsening of the FVC (% pred) and DLco (% pred), while the FEV_1_ (% pred) was significantly increased. *CCRT*, concurrent chemoradiotherapy; *FVC*, functional volume capacity; *FEV1*, forced expiratory volume in 1 s; *DLco*, diffusing capacity of the lung for carbon monoxide

After chemoradiotherapy DLco was decreased by a mean of 12.3% (±10.5) and it was statistically significant (*P* < 0.001). The FVC was also decreased (91.7% vs. 90.3%, *P* = .0.012) but the FEV_1_ was significantly increased after CCRT (82.8% vs. 84.4%, *P* = 0.001) (Fig. 2).

### Postoperative pulmonary complications based on DLco before and after CCRT

**Fig. 3:**
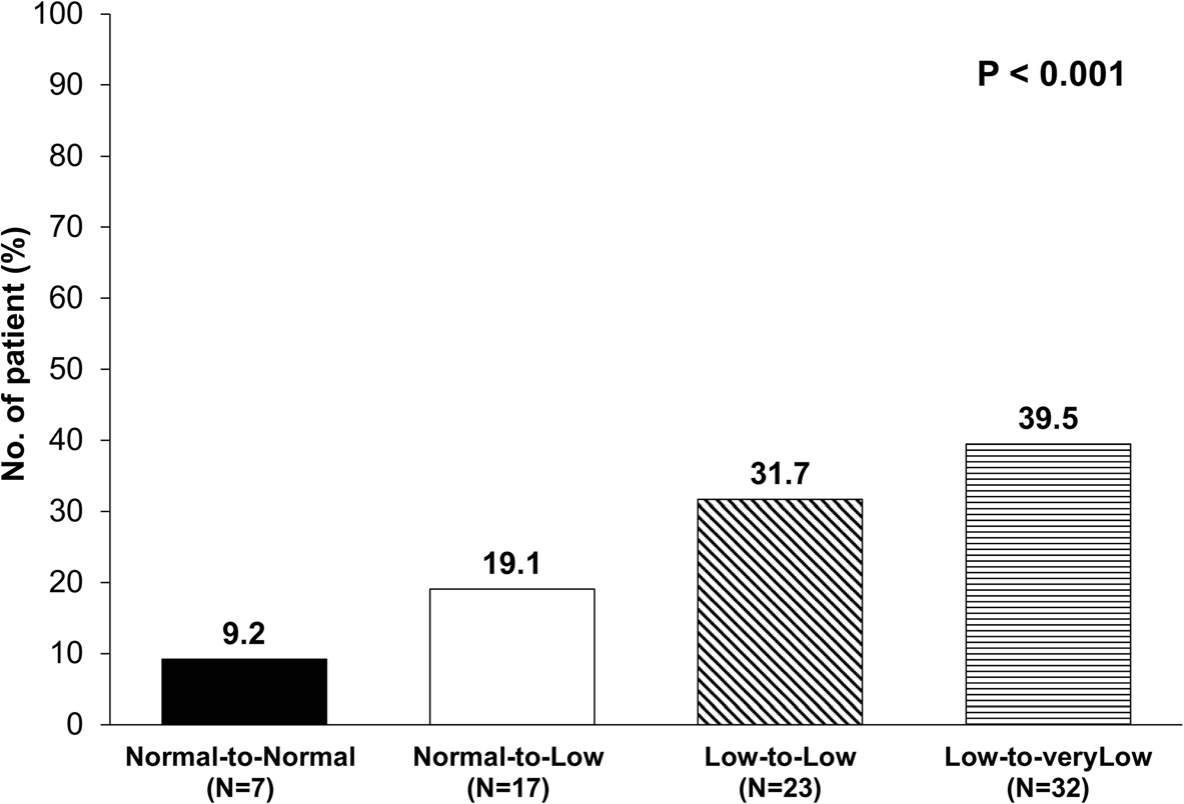
Incidence of PPC according to DLco before and after CCRT. The postoperative pulmonary complications was significantly increased across the four groups based on diffusing lung DLco and the change of DLco after CCRT. *DLco*, diffusing capacity of the lung for carbon monoxide; *CCRT*, concurrent chemoradiotherapy

The details of the pulmonary complication by DLco status was listed in Table 2. The frequency of overall PPC was greater in patients with a low DLco before CCRT compared to those with a normal DLco (35.3% vs. 14.5%, *P* < 0.001). PPC significantly increased across the four groups based on the DLco before and after CCRT (Fig. 3, *P* < 0.001). Major PPC, such as pneumonia/ARDS (*P* < 0.001) and respiratory failure (*P* < 0.001) developed more often in the LVL group. While there was no significant difference in 30-day mortality, 90-day mortality was significantly higher among the LL group (13.3%) and LVL group (13.6%) compared to those of the NN group (1.3%) (*P* = 0.002).

**Table 2:** Incidence of Postoperative Pulmonary Complications (PPCs) by DLco Status

	PPC	Type of the PPC					Mortality	
		ARDS/Pneumonia	Respiratory failure	Air leakage	BPF/empyema	Atelectasis	30-day	90-day
Overall	79 (24.6)	49 (15.3)	23 (7.2)	19 (5.9)	14 (4.4)	10 (3.1)	2 (0.6)	26 (8.1)
DLco at diagnosis								
Normal (*N* = 165)	24 (14.5)	14 (8.5)	4 (2.4)	5 (3.0)	4 (2.4)	4 (2.4)	0 (0)	5 (3.0)
Low (*N* = 156)	55 (35.3)	35 (22.4)	19 (12.2)	14 (9.0)	10 (6.4)	6 (3.9)	2 (1.3)	21 (13.5)
*P* value	<.001	.001	<.001	.024	.081	.533^a^	.234^a^	.001
Change before and after CCRT								
NN: Normal → Normal (*N* = 76)	7 (9.2)	3 (4.0)	0 (0)	1 (1.3)	2 (2.6)	1 (1.3)	0 (0)	1 (1.3)
NL: Normal → Low (*N* = 89)	17 (19.1)	11 (12.4)	4 (4.5)	4 (4.5)	2 (2.3)	3 (3.4)	0 (0)	4 (4.5)
LL: Low → Low (*N* = 75)	23 (31.7)	14 (18.7)	7 (9.3)	7 (9.3)	5 (6.7)	2 (2.7)	1 (1.3)	10 (13.3)
LVL: Low → Very Low (*N* = 81)	32 (39.5)	21 (25.9)	12 (14.8)	7 (8.6)	5 (6.2)	4 (4.9)	1 (1.2)	11 (13.6)
*P for trends*	<.001	<.001	<.001	.030	.148	.255	.260	.002

*ARDS* acute respiratory distress syndrome; *BPF* bronchopleural fistula; *DLco* diffusing capacity of the lung for carbon monoxide; *PPC* postoperative pulmonary complication;

^a^ Fisher’s exact test

Compared to patients with a normal DLco before CCRT, those with a low DLco before CCRT showed a significant increase risk in PPC [Incidence rate ratio (IRR) = 2.14, 95% confidence interval (CI) = 1.36–3.36].

When DLco before and after CCRT were categorized into the four groups, patients in the NL group had no differences in any PPC compared to the NN group. However, the IRR for any PPC, comparing the LL group with the NN group, was 3.03 (95% CI = 1.34–6.89). Furthermore, patients with LVL group showed approximately four-fold increased risk of developing PPC (IRR = 3.78, 95% CI = 1.68–8.49) (Table 3).

**Table 3:** Incidence Rate Ratio (95% Confidence Intervals) for PPCs by DLco Status

	Crude IRR (95% CI)	Model 1 IRR (95% CI)	Model 2 IRR (95% CI)
DLco at diagnosis			
Normal (*N* = 165)	*Reference*	*Reference*	*Reference*
Low (*N* = 156)	2.42 (1.58, 3.72)	2.14 (1.38, 3.30)	2.14 (1.36, 3.36)
*P* value	< .001	.001	.001
Dlco Before And After CCRT			
NN: Normal → Normal (*N* = 76)	*Reference*	*Reference*	*Reference*
NL: Normal → Low (*N* = 89)	2.07 (0.91, 4.74)	2.01 (0.88, 4.60)	2.05 (0.89, 4.73)
LL: Low → Low (*N* = 75)	3.33 (1.52, 7.30)	3.00 (1.34, 6.73)	3.03 (1.34, 6.89)
LVL: Low → Very Low (*N*=81)	4.29 (2.01, 9.14)	3.63 (1.67, 7.88)	3.78 (1.68, 8.49)
*P* for trends	< .001	.004	.005

*CCRT* concurrent chemoradiotherapy; *CI* confidence intervals; *DLco* diffusing capacity of the lung for carbon monoxide; *FEV*_*1*_ forced expiratory volume in one second; *FVC* forced vital capacity; *IRR* incidence rate ratio; *PPC* postoperative pulmonary complication

Model 1: Adjusted for age, sex and type of surgery (lobectomy vs others)

Model 2: Further adjusted for post CCRT airflow limitation (FEV_1_ / FVC < 70%), and post CCRT hemoglobin

**Fig. 4:**
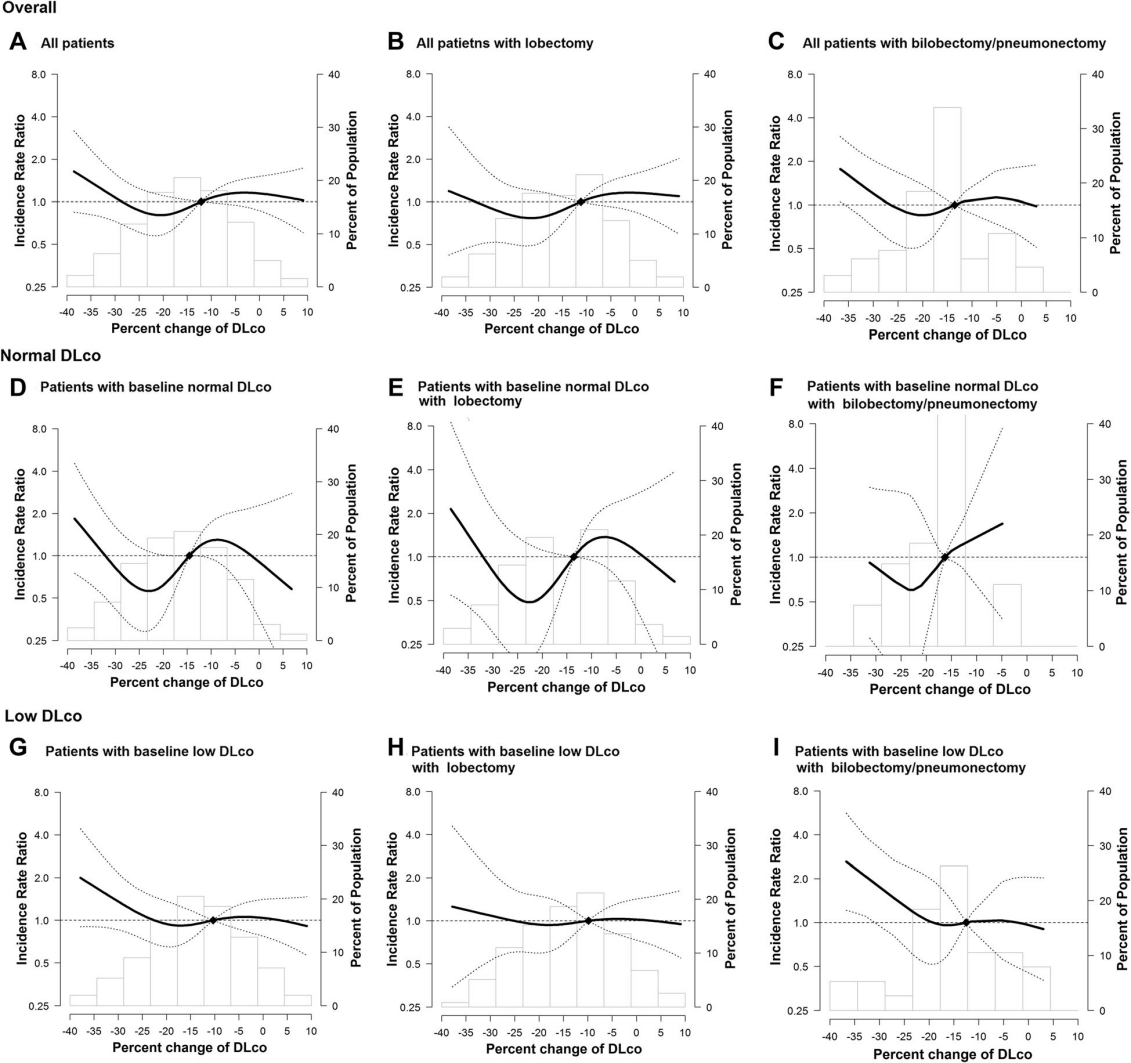
Incidence rate ratio (IRR) for PPC by DLco status and surgical extent. (**a**) All patients. (**b**) All patients with lobectomy. (**c**) All patients with bilobectomy/pneumonectomy. (**d**) Patients with baseline normal DLco. (**e**) Patients with baseline normal DLco with lobectomy. (**f**) Patients with baseline normal DLco with bilobectomy/pneumonectomy. (**g**) Patients with baseline low DLco. (**h**) Patients with baseline low DLco with lobectomy. i) Patients with baseline low DLco with bilobectomy/pneumonectomy. There was a tendency to increase IRR for any postoperative pulmonary complication (PPC) when there was an approximately 20% decrement of DLco after neoadjuvant CCRT. In particular, the risk of PPC after bilobectomy or pneumonectomy sharply increased with greater than 20% of decrement of DLco after neoadjuvant CCRT in patients with low baseline DLco. *DLco*, diffusing capacity of the lung for carbon monoxide; *CCRT*, concurrent chemoradiotherapy

These findings were also consistent after sensitivity analyses among patients who underwent lobectomy (Additional file 1: Table S2). The aIRR for any PPC tended to increase with a decrement of DLco after CCRT. In particular, the risk of PPC after bilobectomy or pneumonectomy sharply increased in patients with a low DLco before CCRT, when they had a greater than approximately 20% decrement of DLco after CCRT (Fig. 4).

## Discussion

In this study with patients with stage IIIA/N2 NSCLC, we found that neoadjuvant CCRT was associated with significant worsening of the DLco, and the risk of PPC was mainly determined by the DLco before CCRT rather than DLco after CCRT. Moreover, we also found that the pronounced change in DLco after neoadjuvant CCRT had a negative impact on PPC after bilobectomy or pneumonectomy, in particular, among patients with a low DLco before CCRT.

While neoadjuvant CCRT followed by surgery improves the oncological outcomes of IIIA/N2 NSCLC [3–6], it increases the risk of postoperative complications [7]. In our study, however, the risk of having PPC was not significantly increased in patients with a normal DLco before CCRT, regardless of deterioration in DLco after CCRT. Moreover, patients with a consistently normal range DLco both before and after CCRT had less than a 10% incidence of PPC, which is comparable to those in early-stage NSCLC patients who had surgery without neoadjuvant treatment [20, 21]. In other words, the results of our study indicate that neoadjuvant CCRT followed by surgery could have acceptable morbidity in patients with a normal DLco both before and after CCRT.

With accounting DLco before and after CCRT, LL group had an approximately 3.0-fold increase in their risk for PPC and LVL group had approximately 3.8-fold increase in their risk for PPC, compared to patients with a consistently normal DLco before and after CCRT. As bilobectomy or pneumonectomy is a very strong risk factor for PPC, we performed sensitivity analyses according to the surgical extent to define the true effects of DLco. When the analyses were restricted to patients with lobectomy, the relative risk for PPC were similar between the LL group and the LVL group supporting that risk of PPC is mainly determined by DLco before CCRT. Nevertheless, remarkable reduction of DLco after neoadjuvant CCRT tended to increase the risk of PPCs when we restricted the analysis to patients with bilobectomy or pneumonectomy who had a low DLco before CCRT. This might be because pneumonectomy is associated with a significant decrease in pulmonary function with anatomical change [22]. In addition, pulmonary and systemic vascular resistance and arteriovenous oxygen difference were more pronounced after pneumonectomy or bilobectomy compared to after lobectomy, leading to a number of potential complications that involve the pulmonary and cardiovascular system [10, 23, 24]. A low DLco before CCRT implicating insufficient remaining healthy lung parenchyma might give an additional effect on increasing PPC risk after bilobectomy or pneumonectomy. Thus, repeated evaluation of DLco would be necessary, particularly in patients with a low DLco before CCRT. A further implication of our study is that definitive CCRT might be considered another option, particularly in patients who have a low DLco before neoadjuvant CCRT and are anticipated to undergo bilobectomy or pneumonectomy.

Our study has several limitations. First, the study used data from a retrospective cohort, not all confounders or outcomes were included in the analysis. However, we were able to adjust major risk factors for PPC. Secondly, as this study was conducted at a referral hospital with comprehensive cancer center, the results of the study might not be generalizable to different settings [25]. Finally, our study could not evaluate the detailed causes of low DLco (e.g., emphysema, interstitial lung abnormalities, or pulmonary vasculopathy). Future study is required to elucidate the impact of the neoadjuvant chemoradiation therapy on each of underlying causes of low DLco. In particular, as the presence of emphysema is a common finding among lung cancer patients even in the patients with normal lung function, emphysema stratification and advanced imaging quantification using Parametric Response Mapping (PRM) on chest computed tomography would be of great value.

## Conclusions

In conclusion, the risk of PPC was mainly associated with DLco before CCRT, and repeated testing would be also helpful for risk assessment, particularly in patients with a low DLco before neoadjuvant CCRT. These findings could provide therapeutically important information, particularly in terms of patient selection for surgery after neoadjuvant CCRT in those with stage IIIA/N2 NSCLC.

## Supplementary information

**Additional file 1: Table S1.** Definitions of the Postoperative Pulmonary Complications. **Table S2.** Incidence Rate Ratio (95% Confidence Intervals) for Postoperative Pulmonary Complications by DLco Status after Lobectomy (*N* = 256)

## Abbreviations

ARDS: Acute respiratory distress syndrome
aRR: Adjusted relative risk
CCRT: Concurrent chemoradiotherapy
CI: Confidence intervals
CT: Computed tomography
DLco: Diffusing capacity of the lung for carbon monoxide
EBUS-TBNA: Endobronchial ultrasound-guided transbronchial needle aspiration
FEV_1_: Forced expiratory volume in 1 s
FVC: Forced vital capacity
IRR: Incidence rate ratio
L: Low
N: Normal
NSCLC: Non-small-cell lung cancer
PET: Positron emission tomography
PPC: Postoperative pulmonary complication
TRT: Thoracic radiation therapy
VL: Very low

## References

[1] Goldstraw P, Crowley J, Chansky K, Giroux DJ, Groome PA, Rami-Porta R, Postmus PE, Rusch V, Sobin L (2007). The IASLC lung Cancer staging project: proposals for the revision of the TNM stage groupings in the forthcoming (seventh) edition of the TNM classification of malignant tumours. J Thorac Oncol.

[2] Goldstraw P, Chansky K, Crowley J, Rami-Porta R, Asamura H, Eberhardt WE, Nicholson AG, Groome P, Mitchell A, Bolejack V (2016). The IASLC lung Cancer staging project: proposals for revision of the TNM stage groupings in the forthcoming (eighth) edition of the TNM classification for lung Cancer. J Thorac Oncol.

[3] Koshy M, Fedewa SA, Malik R, Ferguson MK, Vigneswaran WT, Feldman L, Howard A, Abdelhady K, Weichselbaum RR, Virgo KS (2013). Improved survival associated with neoadjuvant chemoradiation in patients with clinical stage IIIA(N2) non-small-cell lung cancer. J Thorac Oncol.

[4] Katayama H, Ueoka H, Kiura K, Tabata M, Kozuki T, Tanimoto M, Fujiwara T, Tanaka N, Date H, Aoe M (2004). Preoperative concurrent chemoradiotherapy with cisplatin and docetaxel in patients with locally advanced non-small-cell lung cancer. Br J Cancer.

[5] Cerfolio RJ, Maniscalco L, Bryant AS (2008). The treatment of patients with stage IIIA non-small cell lung cancer from N2 disease: who returns to the surgical arena and who survives. Ann Thorac Surg.

[6] Kim HK, Cho JH, Choi YS, Zo JI, Shim YM, Park K, Ahn MJ, Ahn YC, Kim K, Kim J (2016). Outcomes of neoadjuvant concurrent chemoradiotherapy followed by surgery for non-small-cell lung cancer with N2 disease. Lung Cancer.

[7] Kim AW, Boffa DJ, Wang Z, Detterbeck FC (2012). An analysis, systematic review, and meta-analysis of the perioperative mortality after neoadjuvant therapy and pneumonectomy for non-small cell lung cancer. J Thorac Cardiovasc Surg.

[8] Shin S, Park HY, Kim H, Kim HK, Choi YS, Kim J, Um SW, Chung MJ, Kim H, Kwon OJ (2016). Joint effect of airflow limitation and emphysema on postoperative outcomes in early-stage nonsmall cell lung cancer. Eur Respir J.

[9] Pierce RJ, Copland JM, Sharpe K, Barter CE (1994). Preoperative risk evaluation for lung cancer resection: predicted postoperative product as a predictor of surgical mortality. Am J Respir Crit Care Med.

[10] Perentes J, Bopp S, Krueger T, Gonzalez M, Jayet PY, Lovis A, Matzinger O, Ruffieux C, Ris HB, Letovanec I, Peters S (2012). Impact of lung function changes after induction radiochemotherapy on resected T4 non-small cell lung cancer outcome. Ann Thorac Surg.

[11] Leo F, Solli P, Spaggiari L, Veronesi G, de Braud F, Leon ME, Pastorino U (2004). Respiratory function changes after chemotherapy: an additional risk for postoperative respiratory complications?. Ann Thorac Surg.

[12] Cerfolio RJ, Talati A, Bryant AS (2009). Changes in pulmonary function tests after neoadjuvant therapy predict postoperative complications. Ann Thorac Surg.

[13] Rivera MP, Detterbeck FC, Socinski MA, Moore DT, Edelman MJ, Jahan TM, Ansari RH, Luketich JD, Peng G, Monberg M (2009). Impact of preoperative chemotherapy on pulmonary function tests in resectable early-stage non-small cell lung cancer. Chest.

[14] Miller MR, Hankinson J, Brusasco V, Burgos F, Casaburi R, Coates A, Crapo R, Enright P, van der Grinten CP, Gustafsson P (2005). Standardisation of spirometry. Eur Respir J.

[15] American Thoracic Society. Single-breath carbon monoxide diffusing capacity (transfer factor). Recommendations for a standard technique--1995 update. Am J Respir Crit Care Med. 1995;152:2185–98. 10.1164/ajrccm.152.6.8520796 8520796

[16] Park J, Choi I, Park K (1985). Normal predicted standards of single breath carbon monoxide diffusing capacity of lung in healthy nonsmoking adults. Korean J Intern Med.

[17] Harvey BG, Strulovici-Barel Y, Kaner RJ, Sanders A, Vincent TL, Mezey JG, Crystal RG (2015). Risk of COPD with obstruction in active smokers with normal spirometry and reduced diffusion capacity. Eur Respir J.

[18] Pellegrino R, Viegi G, Brusasco V, Crapo RO, Burgos F, Casaburi R, Coates A, van der Grinten CP, Gustafsson P, Hankinson J (2005). Interpretative strategies for lung function tests. Eur Respir J.

[19] Dindo D, Demartines N, Clavien PA (2004). Classification of surgical complications: a new proposal with evaluation in a cohort of 6336 patients and results of a survey. Ann Surg.

[20] Brunelli A, Salati M, Rocco G, Varela G, Van Raemdonck D, Decaluwe H, Falcoz PE (2017). European risk models for morbidity (EuroLung1) and mortality (EuroLung2) to predict outcome following anatomic lung resections: an analysis from the European Society of Thoracic Surgeons database. Eur J Cardiothorac Surg.

[21] Kim ES, Kim YT, Kang CH, Park IK, Bae W, Choi SM, Lee J, Park YS, Lee CH, Lee SM (2016). Prevalence of and risk factors for postoperative pulmonary complications after lung cancer surgery in patients with early-stage COPD. Int J Chron Obstruct Pulmon Dis.

[22] Bolliger CT, Jordan P, Soler M, Stulz P, Tamm M, Wyser C, Gonon M, Perruchoud AP (1996). Pulmonary function and exercise capacity after lung resection. Eur Respir J.

[23] Van Mieghem W, Demedts M (1989). Cardiopulmonary function after lobectomy or pneumonectomy for pulmonary neoplasm. Respir Med.

[24] Smulders SA, Holverda S, Vonk-Noordegraaf A, van den Bosch HC, Post JC, Marcus JT, Smeenk FW, Postmus PE (2007). Cardiac function and position more than 5 years after pneumonectomy. Ann Thorac Surg.

[25] Bach PB, Cramer LD, Schrag D, Downey RJ, Gelfand SE, Begg CB (2001). The influence of hospital volume on survival after resection for lung cancer. N Engl J Med.

