# Supplementary material for: Impact of diffusing lung capacity before and after neoadjuvant concurrent chemoradiation on postoperative pulmonary complications among patients with stage IIIA/N2 non-small-cell lung cancer
Source: Respir Res. 2020 Jan 10;21:13. doi: 10.1186/s12931-019-1254-0 (PMC6954564; doi:10.1186/s12931-019-1254-0)
Supplement: Supplementary file 1 — Additional file 1: Table S1. Definitions of the Postoperative Pulmonary Complications. Table S2. Incidence Rate Ratio (95% Confidence Intervals) for Postoperative Pulmonary Complications by DLco Status after Lobectomy (N = 256) [file 12931_2019_1254_MOESM1_ESM.doc]

**e-Table 1] Definitions of the Postoperative Pulmonary Complications**

| **Complication** | **Definition** |
| --- | --- |
| **Pneumonia** | Patient received antibiotics for a suspected respiratory infection  Newly appeared pulmonary infiltration on chest X-ray  And met at least one of the following criteria.  - New onset or changed sputum  - Fever  - Leukocytosis (>12000/u)  - Documented pathogen on sputum culture |
| **Acute respiratory distress syndrome**  **(ARDS)** | Patient met at least one of the following criteria.   - PaO2 <60 mmHg on room air - PaO2 to inspired oxygen fraction < 300 - Arterial oxyhemoglobin saturation measured with pulse oximetry <90%   And newly appeared pulmonary infiltration on chest X-ray |
| **Respiratory failure** | Indicate whether the patient experienced respiratory failure in the postoperative period requiring mechanical ventilation and/or reintubation |
| **Significant atelectasis** | Patient who required therapeutic bronchoscopy or reintubation to control the atelectasis.* |
| **Empyema** | Pus or infected fluid collects in a pleural space |
| **Bronchopleural fistula (BPF)** | Abnormal passage that develops between bronchus and the pleura which was demonstrated on chest computed tomography scan or bronchoscopy |
| **Prolonged air leakage** | Air leakage was lasting for more than 5 days or requiring pleurodesis or reinsertion of chest tube. |

***** Patients accompanying pneumonia or ARDS were not included.

**e-Table 2] Incidence Rate Ratio (95% Confidence Intervals) for Postoperative Pulmonary Complications**

**by DLco Status after Lobectomy (N = 256)**

| **Change of DLco, % of the predicted value** | **Crude**  **IRR (95% CI)** | **Model 1**  **IRR (95% CI)** | **Model 2**  **IRR (95% CI** |
| --- | --- | --- | --- |
| **DLco at diagnosis** |  |  |  |
| Normal (N = 138) | *Reference* | *Reference* | *Reference* |
| Low (N = 118) | 3.12 (1.85, 5.44) | 2.81 (1.62, 4.90) | 2.79 (1.54, 5.05) |
| P value | <.001 | <.001 | .001 |
| **Change before and after CCRT** |  |  |  |
| NN: Normal → Normal (N = 70) | *Reference* | *Reference* | *Reference* |
| NL: Normal → Low (N = 68) | 2.06 (0.74, 5.72) | 2.19 (0.78, 6.14) | 2.20 (0.79, 6.16) |
| LL: Low → Low (N = 55) | 4.58 (1.81, 11.58) | 4.43 (1.74, 11.30) | 4.41 (1.69, 11.51) |
| LVL: Low → Very Low (N = 63) | 4.89 (1.97, 12.16) | 4.39 (1.74, 11.09) | 4.42 (1.64, 11.90) |
| P for trend | <.001 | <.001 | <.001 |

CCRT = concurrent chemoradiotherapy; CI = confidence intervals; DLco = diffusing capacity of the lung for carbon monoxide; FEV_1_ = forced expiratory volume in one second; FVC = forced vital capacity; IRR =incidence relative risk; PPC = postoperative pulmonary complications

Model 1: Adjusted for age, sex and type of surgery (lobectomy vs others)

Model 2: Further adjusted for post CCRT airflow limitation (FEV_1_ / FVC < 70%), and post CCRT hemoglobin
